# Concurrent predictors of an immune responsive tumor microenvironment within tumor mutational burden-high breast cancer

**DOI:** 10.3389/fonc.2023.1235902

**Published:** 2023-08-11

**Authors:** Sarah Sammons, Andrew Elliott, Romualdo Barroso-Sousa, Saranya Chumsri, Antoinette R. Tan, George W. Sledge, Sara M. Tolaney, Evanthia T. Roussos Torres

**Affiliations:** ^1^Department of Medical Oncology, Dana-Farber Cancer Institute, Boston, MA, United States; ^2^Breast Oncology Program, Dana-Farber Brigham Cancer Center, Boston, MA, United States; ^3^Harvard Medical School, Boston, MA, United States; ^4^Clinical and Translational Research, Caris Life Sciences, Phoenix, AZ, United States; ^5^Department of Oncology, Dasa Institute for Education and Research (IEPD), Brasilia, Brazil; ^6^Dasa Oncology/Hospital Brasilia, Brasilia, Brazil; ^7^Department of Hematology Oncology and Department of Cancer Biology, Mayo Clinic, Jacksonville, FL, United States; ^8^Levine Cancer Institute, Atrium Health, Charlotte, NC, United States; ^9^ Division of Oncology, Department of Medicine, Keck School of Medicine, Norris Comprehensive Cancer Center, University of Southern California, Los Angeles, CA, United States

**Keywords:** breast cancer, tumor mutational burden, genetic profiling, microenvironment, immune checkpoint inhibitors

## Abstract

**Background:**

Data supporting high tumor mutational burden (TMB-H) as a lone biomarker for an immune-responsive tumor microenvironment (TME) in metastatic breast cancer (MBC) are weak, yet tumor agnostic approval in TMB-H advanced tumors provides immune checkpoint inhibition (ICI) as a clinical option. We evaluated concurrent predictors of immune-responsive and non-responsive TME within MBC.

**Methods:**

Tumor samples from patients with MBC (N=5621) were analyzed by next-generation sequencing of DNA (592-gene panel or whole exome) and RNA (whole transcriptome) at Caris Life Sciences (Phoenix, AZ). TMB-H threshold was set to ≥ 10 muts/Mb. PDL-1 was evaluated using SP142 antibody. Gene expression profiling and RNA deconvolution were used to estimate immune and stromal cell population abundance in the TME, and transcriptomic signature of immunotherapy response (T cell-inflamed score).

**Results:**

461 (8.2%) TMB-H MBC samples were identified. Consistent with prior studies, TMB-H tumors exhibited significant dMMR/MSI-H enrichment (7 vs. 0%, p<0.0001) and PD-L1+ expression (36 vs. 28%, p<0.05) compared to TMB-L. Across all samples, T cell-inflamed scores were weakly correlated with TMB. TMB-H was not associated with significantly increased immune responsive cell types (CD8+ T-cells, NK cells, or B cells) or immune response gene signatures (e.g. antigen presentation), yet positive trends were observed, while immunosuppressive fibroblasts were significantly decreased in TMB-H tumors (0.84-fold change compared to TMB-L, P<0.05). HR+/HER2- breast cancer was the only subtype in which TMB-H tumors exhibited increased T cell-inflamed scores vs. TMB-L. Concurrent PD-L1+ or dMMR/MSI-H with TMB-H was associated with high T cell-inflamed scores in both HR+/HER2- and TNBC. Among several associated biomarkers, *B2M* mutations and *CD274* amplifications were positively associated with T-cell inflamed scores in TMB-H tumors; *CDH1* and *ERBB2* mutations were negatively associated.

**Conclusion:**

High TMB alone does not strongly correlate with immune infiltrate or immune-related gene signatures in MBC. TMB-H predicts T-cell inflamed signature compared to TMB-L in HR+/HER2- tumors only. Along with MSI-H and PD-L1+, several biomarkers, including *B2M* mutation and *CD274* amplification, may help predict ICI benefit amongst TMB-H tumors. Co-occurring biomarkers within TMB-H breast cancer warrant evaluation in larger cohorts for response or resistance to ICI to develop composite predictive biomarkers in MBC.

## Introduction

Data supporting high tumor mutational burden (TMB-H) as a lone biomarker for an immune-responsive tumor microenvironment (TME) in metastatic breast cancer (MBC) is weak, yet the tumor agnostic approval of immune checkpoint inhibitors (ICI) in TMB-H advanced tumors makes this an option in the clinic. The U.S. Food and Drug Administration (FDA) approved pembrolizumab on June 16, 2020, for the advanced TMB-H (≥10 mutations/megabase (mut/Mb), as determined by an FDA-approved test) solid tumors that have progressed following prior treatment with no satisfactory alternative treatment options. This approval was based on an overall response rate of 29% (95% CI, 21-39) in the subset of patients with TMB-H solid tumors (*n* = 102) spanning nine different tumor types enrolled in a multicenter single-arm trial KEYNOTE-158 (1). There were no MBC patients in this trial, making extrapolation to this population impossible. In pooled meta-analyses, TMB does not predict survival in MBC (2).

The TAPUR clinical trial included a TMB-H MBC cohort that enrolled 28 patients with TMB ranging from 9-37 mut/Mb by Foundation Medicine CDX, who were enrolled to receive pembrolizumab monotherapy every 3 weeks (3). The overall response rate (ORR) was 21% (95% confidence interval (CI), 8 to 41), and the median progression-free survival (PFS) was 10.6 weeks (95% CI, 7.7 to 21.1). Though this is a respectable ORR in heavily pretreated MBC for therapy with tolerable safety, most TMB-H patients will not derive benefit from ICI monotherapy, and further biomarkers within TMB-H MBC to predict an immune hot TME are needed.

We sought to further evaluate concurrent predictors of an immune-responsive or non-responsive TME within TMB-H MBC.

## Materials and methods

### Patient samples/study cohort

Formalin-fixed paraffin-embedded (FFPE) samples from patients with breast cancer (n=5621) were submitted by various academic and community cancer institutes, predominately in the United States, to a commercial CLIA-certified laboratory for molecular profiling (Caris Life Sciences, Phoenix, AZ). The present study was conducted in accordance with the guidelines of the Declaration of Helsinki, Belmont Report, and U.S. Common Rule. In compliance with policy 45 CFR 46.101(b), this study was conducted using retrospective, de-identified clinical data, patient consent was not required, and the study was considered IRB exempt.

### Next-generation sequencing (NGS)

NGS of 592 cancer-relevant genes was performed on genomic DNA isolated from formalin-fixed paraffin-embedded (FFPE) tumor samples using the NextSeq platform (Illumina, Inc., San Diego, CA, USA). Matched normal tissue or germline DNA was not sequenced. A custom-designed SureSelect XT assay was used to enrich exonic regions of 592 whole-gene targets (Agilent Technologies, Santa Clara, CA, USA). All variants were detected with >99% confidence based on allele frequency and amplicon coverage, with an average sequencing depth of coverage of >500 and an analytic sensitivity threshold established of 5% for variant calling. Prior to molecular testing, tumor enrichment was achieved by harvesting targeted tissue using manual microdissection techniques. Genomic variants were classified by board-certified molecular geneticists according to criteria established by the American College of Medical Genetics and Genomics (ACMG). When assessing mutation frequencies of individual genes, ‘pathogenic’ and ‘likely pathogenic’ were counted as mutations, while ‘benign’, ‘likely benign’ variants, and ‘variants of unknown significance’ were excluded.

RNA Whole Transcriptome Sequencing (WTS) uses a hybrid-capture method to pull down the full transcriptome from FFPE tumor samples (using the Agilent SureSelect Human All Exon V7 bait panel (Agilent Technologies, Santa Clara, CA, USA) and the Illumina NovaSeq platform (Illumina, Inc., San Diego, CA, USA). FFPE specimens underwent pathology review to discern the percent tumor content and tumor size; a minimum of 20% tumor content in the area for microdissection was required to enable enrichment and extraction of tumor-specific RNA. A Qiagen RNA FFPE tissue extraction kit was used for extraction, and the RNA quality and quantity were determined using the Agilent TapeStation. Biotinylated RNA baits were hybridized to the synthesized and purified cDNA targets, and the bait-target complexes were amplified in a post-capture PCR reaction. The resultant libraries were quantified and normalized, and the pooled libraries were denatured, diluted, and sequenced. Raw data were demultiplexed using the Illumina DRAGEN FFPE accelerator. FASTQ files were aligned with STAR aligner (Alex Dobin, release 2.7.4a GitHub). A full 22,948-gene dataset of expression data was produced by the Salmon, which provided fast and bias-aware quantification of transcript expression BAM files from STAR aligner (4), and were further processed for RNA variants using a proprietary custom detection pipeline. The reference genome used was GRCh37/hg19, and analytical validation of this test demonstrated ≥97% Positive Percent Agreement (PPA), ≥99% Negative Percent Agreement (NPA), and ≥99% Overall Percent Agreement (OPA) with a validated comparator method.

### RNA signatures

T cell-inflamed scores were defined by an 18-gene signature, with scores calculated as the weighted sum of log2-transformed gene expression values using previously reported coefficients (5). The Microenvironment Cell Populations (MCP)-counter tool was used to assess the relative abundance of immune and stromal cells in the tumor microenvironment (6, 7).

### Immunohistochemistry (IHC)

IHC was performed on FFPE sections of glass slides. Slides were stained using the Agilent DAKO Link 48 (Santa Clara, CA, USA) automated platform and staining techniques, per the manufacturer’s instructions, and were optimized and validated per CLIA/CAP and ISO requirements. Staining was scored for intensity (0 = no staining; 1+ = weak staining; 2+ = moderate staining; 3+ = strong staining) and staining percentage (0–100%). Positive expression of immune cell (IC) PD-L1 (SP142), tumor cell ESTROGEN RECEPTOR (ER), and tumor cell PROGESTERONE RECEPTOR (PR) was defined as ≥1+ stain intensity and ≥1% of cells stained. Positive HER2 expression was determined according to the 2018 ASCO-CAP guidelines (8).

### Tumor mutational burden (TMB)

TMB was measured by counting all non-synonymous missense, nonsense, in-frame insertion/deletion, and frameshift mutations found per tumor that had not been previously described as germline alterations in dbSNP151, Genome Aggregation Database (gnomAD) databases, or benign variants identified by Caris’s geneticists. TMB-H was defined by a threshold of ≥10 mutations per megabase (mut/MB) based on the KEYNOTE-158 pembrolizumab trial (1).

### Mismatch repair/microsatellite instability

Deficient mismatch repair/microsatellite instability-high (dMMR/MSI-H) was determined by a combination of IHC (MLH1, M1 antibody; MSH2, G2191129 antibody; MSH6, 44 anti-body; and PMS2, EPR3947 antibody (Ventana Medical Systems, Inc., Tucson, AZ) and NGS (>2800 target microsatellite loci were examined and compared to the reference genome hg19 from the University of California, Santa Cruz (UCSC) Genome Browser database). The platforms generated highly concordant results as previously reported (9) and in the rare cases of discordant results, the status was determined by IHC.

### Statistical analyses

All statistical analyses were performed with JMP V13.2.1 (SAS Institute, Cary, NC, USA). Continuous data were assessed using a Mann-Whitney U test, and categorical data were evaluated using Chi-square or Fisher’s exact test, where appropriate. P-values were adjusted for multiple hypothesis testing using the Benjamini-Hochberg procedure, unless noted as exploratory (not adjusted).

## Results

### Clinical and molecular characteristics associated with TMB-H breast cancer

Comprehensive molecular profiles of breast cancer patient samples (N=5621) were analyzed from various cancer institutes, predominantly within the United States. Samples were stratified into TMB-H (N=461, 8.2%) and TMB-Low cohorts based on a threshold of ≥10 mut/MB (**Table 1**). Utilizing different cut-offs, 4% of patients had a TMB ≥ 14 and 1.81% had TMB ≥ 20. Compared to TMB-Low tumors, the TMB-H cohort had an increased median age (64 vs. 60 years, p<0.0001) and a greater proportion of distant metastatic tumor biopsy sites compared to breast biopsy specimens (78.3% vs. 60.3%, p<0.0001). As previously described, TMB-H tumors were more likely to be invasive lobular than invasive ductal carcinoma (31.8% vs. 11.3%, p<0.0001) (10). The distribution of receptor subtypes (HR+/HER2-, HR-/HER2-, HR+/HER2+, HR-/HER2+) was similar between TMB-H and TMB-low cohorts.

**Table 1:** Overall cohort characteristics and of TMB-H and TMB-L cohorts.

Characteristic	Overall	TMB-H	TMB-L	P-value
Samples, N (%)	5621 (100%)	461 (8.2%)	5160 (91.8%)	---------
Median Age, years - Range	60 19-90+	64 26-90+	60 19-90+	< 0.0001
Gender, N (%) - Female - Male	5572 (99.1%) 49 (0.9%)	458 (99.3%) 3 (0.7%)	5114 (99.1%) 46 (0.9%)	0.59
Biopsy site - Primary - Metastatic	2115 (37.6%) 3506 (62.4%)	100 (21.7%) 361 (78.3%)	2015 (39.1%) 3145 (60.9%)	< 0.0001
Histology, N (%) - Ductal - Lobular - Metaplastic - Mixed - [NOS]	2273 (40.4%) 349 (6.2%) 85 (1.5%) 58 (1.0%) [2856]	116 (65.9%) 56 (31.8%) 2 (1.1%) 2 (1.1%) [285]	2157 (83.3%) 293 (11.3%) 83 (3.2%) 56 (2.2%) [2571]	< 0.0001
Receptor Subtypes, N (%) - HR+/HER2+ - HR+/HER2- - HR-/HER2+ - Triple Negative - [Unclear]	266 (5.3%) 3087 (61.6%) 179 (3.6%) 1476 (29.5%) [613]	22 (5.4%) 242 (59.7%) 14 (3.5%) 127 (31.4%) [56]	244 (5.3%) 2845 (61.8%) 165 (3.6%) 1349 (29.3%) [557]	0.85

TMB-H, high tumor mutational burden; TMB-L, low tumor mutational burden; NOS, not otherwise specified; HR, hormone receptor; HER2, human epidermal growth factor receptor 2.

### TMB-H is a poor predictor of inflamed tumor microenvironments (TMEs) in breast cancer

**Figure 1:**
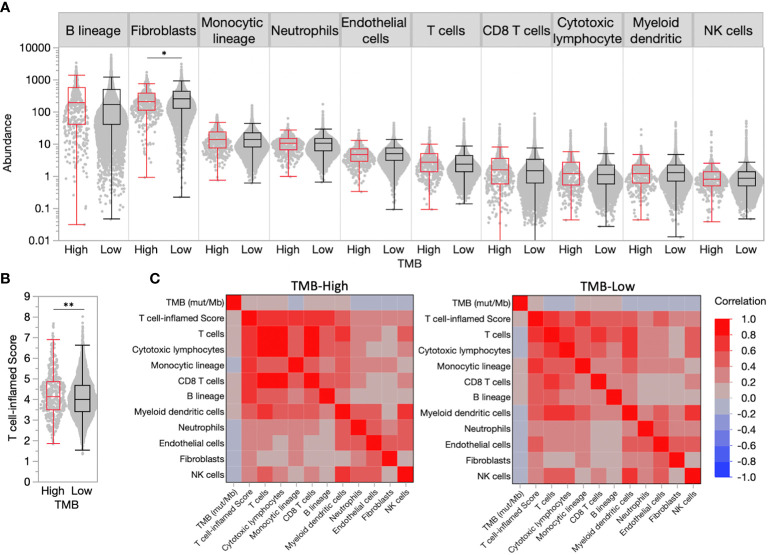
High tumor mutational burden (TMB-H) is a poor predictor of inflamed tumor microenvironments (TMEs) in breast cancer. **(A)** TME immune and stromal cell population abundance in TMB-H and low tumor mutational burden (TMB-L) tumors. **(B)** Distribution of T cell-inflamed scores in TMB-H and TMB-L tumors. **(C)** Correlation matrix for TMB, T cell-inflamed scores, and immune/stromal cell population abundance in TMB-H and TMB-L tumors. *P<0.05, **P<0.01.

Despite the FDA approval of pembrolizumab for the treatment of adult and pediatric patients with unresectable or metastatic TMB-H solid tumors, not all TMB-H tumors will respond to therapy, suggesting additional predictive biomarkers are needed. We estimated immune and stromal cell population abundance in breast cancer TMEs using the MCP-Counter tool and observed similar distributions in TMB-H and TMB-L tumors for most cell populations (**Figure 1A**). While TMB-H tumors have slightly increased median abundance of pro-immune cell types (e.g. T cells, not significant), presumably immunosuppressive fibroblasts were significantly decreased in TMB-H tumors (0.84-fold change compared to TMB-L, P<0.05).

A transcriptional ‘T cell-inflamed’ score, which was previously demonstrated to predict response to ICI therapy in all tumor types (5), was significantly increased in TMB-H tumors compared to TMB-Low (4.15 vs. 4.02, P<0.01), though the score distributions largely overlapped (**Figure 1B**), and correlated with immune and stromal cell population abundance in both TMB-H and TMB-L tumors (**Figure 1C**). Similar to individual TME cell populations, the T cell-inflamed score was weakly correlated with the number of muts/Mb. Tumors with very high TMB > 20 were very limited in our population making true assessments of the immune TME in these patients challenging.

### PD-L1 positivity and Microsatellite instability are enriched in TMB-H tumors and further predict an inflamed tumor microenvironment

**Figure 2:**
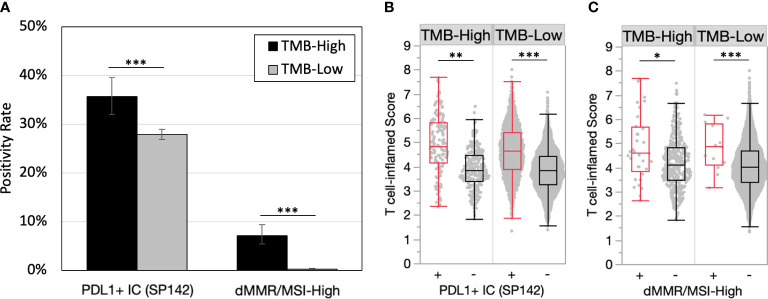
PD-L1 positivity and microsatellite instability are enriched in TMB-H tumors and predict inflamed TMEs. **(A)** PD-L1+ immune cells **(IC)** (SP142) and dMMR/MSI-High positivity rates in TMB-H and TMB-L tumors. **(B-C)** T cell-inflamed scores in by PD-L1+ IC (SP142) **(B)** and dMMR/MSI-High status **(C)**. *P<0.05, **P<0.01, ***P<0.001.

PD-L1 expression on tumor immune cells is a consistent biomarker of ICI response in advanced/metastatic first-line triple-negative breast cancer (TNBC) (11–13). PD-L1 expression on immune cells (PD-L1+ IC [SP142]) was more common in TMB-H versus TMB-Low tumors (35.7% vs. 27.9%, p<0.001) (**Figure 2A**). PD-L1 evaluation by Dako 22C3 combined positive score was not available in this dataset. dMMR/MSI-High, a known mechanism of tumor hypermutation, is another tumor agnostic FDA-approved biomarker for ICI. In the entire cohort, dMMR/MSI-High status was rare with an overall frequency of 0.8% (46/5570) of all breast tumors. dMMR/MSI-High status (7.2% vs. 0.3%, p<0.001) was almost exclusively found in TMB-H tumors. The median TMB of TMB-H/MSI-High was 24 mut/Mb versus 13 mut/Mb TMB-H/MSI stable. Greater differences in median T cell-inflamed scores were observed when samples were further stratified by PD-L1+ IC and dMMR/MSI-High status (**Figures 2B, C**). Tumors that were TMB-H and PD-L1+ had significantly higher T-cell inflamed scores than TMB-H PD-L1- tumors. Interestingly, this was true in both TMB-H and TMB-L cohorts suggesting that immune responsive TME’s exist in TMB-L tumors with PD-L1+.

### TMEs vary across breast cancer receptor subtypes and histologies

**Figure 3:**
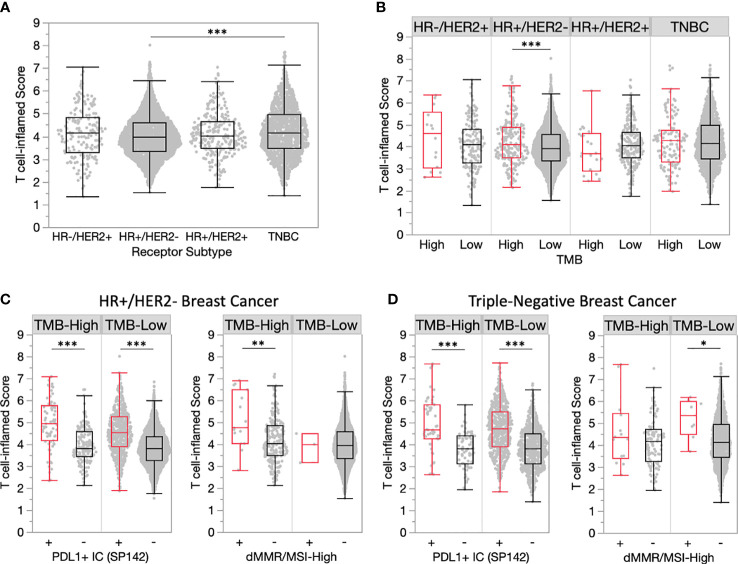
TMEs vary across breast cancer receptor subtypes. **(A)** T cell-inflamed scores in receptor subtype subgroups. **(B)** T cell-inflamed scores in TMB-H and TMB-L tumors by receptor subtype. **(C-D)** T cell-inflamed scores in TMB-H and TMB-L HR+/HER2- **(C)** and triple-negative **(D)** subgroups further stratified by PD-L1+ immune cells **(IC)** (SP142) and dMMR/MSI-High status. *P<0.05, **P<0.01, ***P<0.001.

Among breast cancer receptor subtypes, median T cell-inflamed scores were highest among TNBC samples, which were significantly increased compared to HR+/HER2- samples that exhibited the lowest median score (4.17 vs. 3.96, P<0.001) (**Figure 3A**). While TMB-H HR+/HER2- samples had significantly increased T cell-inflamed scores compared to TMB-L, scores in other receptor subtypes were not significantly different when stratified by TMB status (**Figure 3B**). Similar to the overall trend, T cell-inflamed scores were increased HR+/HER2- and TNBC samples further stratified by PD-L1+ IC status in both TMB-H and TMB-L subgroups, while scores associated with dMMR/MSI-High status varied by receptor subtype (**Figures 3C, D****)**.

**Figure 4:**
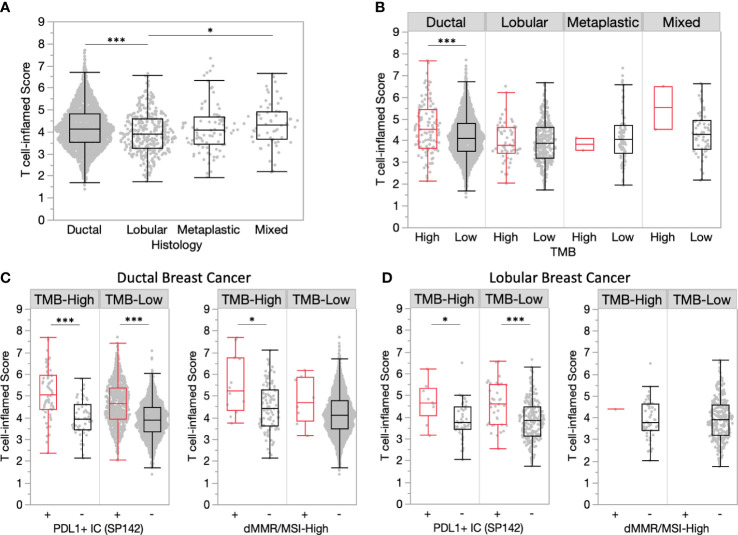
Ductal tumors have more inflamed TMEs compared to lobular tumors. **(A)** T cell-inflamed scores in ductal and lobular subgroups. **(B)** T cell-inflamed scores in TMB-H and TMB-L tumors by histology. **(C-D)** T cell-inflamed scores in TMB-H and TMB-L ductal **(C)** and lobular **(D)** subgroups further stratified by PD-L1+ immune cells **(IC)** (SP142) and dMMR/MSI-High status. *P<0.05, ***P<0.001.

Comparison of histological subtypes found significantly increased T cell-inflamed scores in ductal vs. lobular tumors (4.14 vs. 3.90, P<0.001) (**Figure 4A**). TMB-H ductal tumors had significantly increased T cell-inflamed scores compared to TMB-L, yet this was not observed in other histological subtypes (**Figure 4B**). PDL1+ immune cells within ductal and lobular tumors were consistently associated with significantly increased T cell-inflamed scores regardless of TMB status (**Figures 4C, D**). A similar trend was observed for dMMR/MSI-High ductal tumors, while lobular tumors were rarely dMMR/MSI-High.

### Biomarker association with inflamed tumor microenvironments

**Figure 5:**
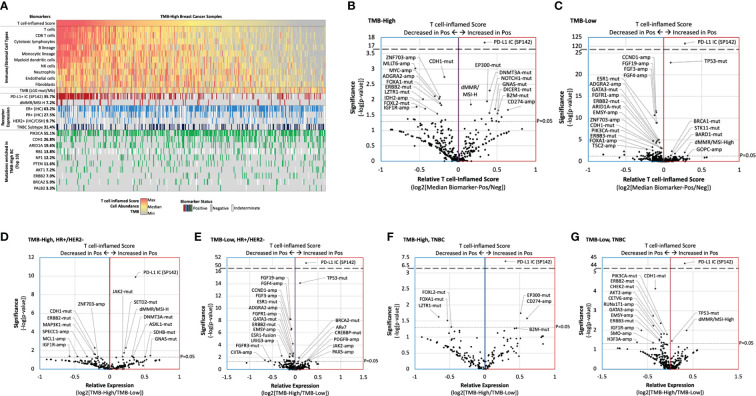
Biomarker association with inflamed tumor microenvironments. **(A)** Oncoprint of TMB-H breast cancer T cell-inflamed score immune/stromal cell population abundance, and key biomarkers. **(B-G)** Relative T cell-inflamed score according to biomarker status (ratio of median biomarker-positive [Pos]/negative [Neg]) in TMB-H **(B, D, F)** and TMB-L samples **(C, E, G)** for the overall cohort (B,C), HR+/HER2- samples **(D, E)**, and TNBC samples **(F, G)**.

To identify new predictive biomarkers of inflamed tumor microenvironments, we compared T cell-inflamed scores in TMB-H and TMB-L cohorts stratified by biomarker status (mutation, amplification, fusion, etc). Consistent with our initial analysis, PD-L1+ IC was associated with higher T cell-inflamed scores in TMB-H tumors, while scores associated with many of the most commonly altered biomarkers were much more variable (**Figure 5A**). However, several other biomarkers were associated with significantly higher or lower scores, including mutations in *CDH1* and *ERBB2* that associated with lower T cell-inflamed scores in TMB-H tumors (**Figure 5B**). This is of interest as *CDH1* mutations are strongly present in lobular breast cancer and is consistent with lower scores in lobular compared to ductal tumors. *ZNF703* and *ADGRA2* copy number amplifications were associated with lower T cell-inflamed scores, regardless of TMB status (**Figures 5B, C**). Several alterations were associated with differences in T cell-inflamed scores only in TMB-H or TMB-Low cohorts. Interestingly, in TMB-H tumors, increased T cell-inflamed scores were associated with mutations in *B2M (Beta-2 microglobulin)*, a scaffolding protein essential for MHC-I complex formation and peptide presentation. *CD274* (PDL-1) amplification was also associated with T cell-inflamed score in TMB-H tumors.

We further evaluated TMB-H vs TMB-L T cell-inflamed scores in HR+/HER2- (**Figures 5D, E**) and TNBC subgroups (**Figures 5F, G**). Many biomarkers were significantly associated with higher or lower T cell-inflamed scores in a receptor subtype-dependent manner. For example, while dMMR/MSI-High and *SETD2* mutations were associated with higher scores and *CDH1* mutations were associated with lower scores in TMB-H HR+/HER2- samples, *EP300* mutations and *CD247* amplifications were associated with higher scores and *LZTR1* mutations were associated with lower scores in TMB-H TNBC samples.

## Discussion

Tumor mutational burden is an overall poor predictor of ICI response in metastatic breast cancer. The TAPUR clinical trial is the largest series to evaluate single agent immunotherapy in TMB-H MBC defined as ≥ 9 mut/Mb by Foundation Medicine CDX. The ORR to single agent pembrolizumab was 21% (95% CI, 8 to 41) and the median PFS was 10.6 weeks (95% CI, 7.7 to 21.1) (3). However, TMB as a continuous variable did not predict response. More recently, the NIMBUS clinical trial evaluated the efficacy of immunotherapy combination, ipilimumab and nivolumab, in TMB-H (defined as ≥ 9 mut/Mb) HER2-negative MBC (14). After a median follow-up of 10 months, the ORR was 16.7%, though the median duration of response has not been reached and 3 patients were progression-free for at least 15 months. The median PFS and overall survival (OS) was respectively 1.4 (95% CI 1.3 - 9.5) months and 8.8 (95% CI 4.2 - not reached). Response rate in patients with TMB ≥ 14 muts/Mb was 60%, suggesting the ultra-high TMB patients may have an immune responsive TME. Given the known toxicities of ICI, identification of biomarkers within TMB-H tumors to predict ICI response and immune responsive TME would be of particular importance.

In a large cohort of 5621 breast cancer tumors, we identified 461 (8.2%) TMB-H tumors and examined concurrent predictive biomarkers of an immune-inflamed TME to assess predictors of immune checkpoint blockade (ICB) response. RNA signatures hold promise as biomarkers of immunotherapy response across solid tumor malignancies. We used a well validated T cell-inflamed scores defined by an 18-gene signature to select tumors with an immune responsive TME within this cohort (5). This immune signature has correlated strongly with response to ICI in solid tumors. The T-cell inflamed score was significantly increased in TMB-H tumors compared to TMB-Low (4.15 vs. 4.02, P<0.01), though the score distributions largely overlapped (**Figure 1B**) indicating weak association. TMB-H was a biomarker of T-cell inflamed score within the HR+, HER2- negative subtype but not in HER2+ or triple negative tumors. This is of particular interest as nearly ¾ of all breast cancers are HR+, HER2-. In unselected HR+/HER2- breast cancer, immune checkpoint inhibition has not been effective (15, 16). Our data support the use of TMB as a biomarker of ICI response in future prospective clinical trials of HR+, HER2- MBC.

We then assessed the impact of known biomarkers of immune response in breast cancer and solid tumors within TMB-H breast cancer and found that PD-L1 positivity and microsatellite instability were enriched in TMB-H tumors and predicted inflamed TMEs. This finding was true regardless of tumor subtype (HR+ and TNBC) and histology (ductal and lobular). These findings are particularly clinically relevant as commercially available next generation sequencing tests routinely report PD-L1 and dMMR/MSI-H status along with TMB. A logical next step to this analysis would be to assess ICI responses in patients with TMB-H and PDL-1+ tumors in prospective or retrospective cohorts. One limitation of this study is the use of PD-L1 testing using the Ventana SP142 assay on tumor immune cells, which is no longer used in United States clinical practice. These findings should be repeated using diverse PD-L1 assays.

Lobular breast cancer encompasses about 10% of all breast tumors with increasing incidence in recent decades (17). Several studies have shown that TMB-H lobular tumors have higher TMB than ductal tumors, making immunotherapy an appealing strategy (18). Inactivating *CDH1* mutations are found in 53% of lobular breast cancers in the literature (19) and have higher median TMB than ductal tumors (18). In this analysis, lobular tumors had significantly lower T-cell inflamed scores than ductal tumors. Furthermore, *CDH1* mutations were associated with lower T-cell inflamed score within TMB-H tumors. T-cell inflamed scores in lobular tumors were similar between TMB-H and TMB-L. These data suggest that neither lobular histology nor the composite of lobular and TMB-H will be strong enough predictors of an immune responsive tumor microenvironment. PD-L1 positivity did still enrich for a higher immune TME within lobular tumors, suggesting PD-L1+ lobular BC could be a better predictor of ICI response. The multicenter GELATO-trial (NCT03147040) evaluated patients with metastatic lobular breast cancer treated with induction carboplatin followed by atezolizumab (PD-L1 inhibitor). Four (4/21) patients with triple negative disease had a partial response to treatment (20) without any responses reported in the HR+ patients, suggesting ICI is not a promising strategy in unselected lobular tumors.

Lastly, our analysis showed that *B2M* mutations and *CD274* amplifications were associated with a strong T-cell inflamed score within TMB-H tumors and not TMB-Low tumors, which was also observed in TNBC but not HR+/HER2- subgroups. Recent data suggest that somatic *B2M* mutations are associated with a higher load of neoantigens for MHC-I presentation (21), which could lead to T cell recognition in the setting of ICI. Programmed death ligand-1 (PD-L1) is encoded by the *CD274* gene is a target for both PDL-1 and PD-1 inhibitors. Although PDL-1/CD274 amplification in solid tumors is rare, it has been linked to ICI response in small series (22). Furthermore, in the randomized phase II SAFIR02-BREAST IMMUNO trial, durvalumab was studied as maintenance therapy after chemotherapy induction in MBC patients, and in an exploratory analyses of TNBC patients, durvalumab efficacy was limited to those with CD274 gain/amplification (23).

There are several limitations of this analysis. The lack of matched treatment and response data limits our ability to determine potential therapy-induced effects on the TME signatures evaluated, as well as limiting the evaluation of immune-related signatures and co-alterations as predictive biomarkers of response to therapy. Additionally, as bulk tumor sequencing approaches do not allow for robust characterization of cell type-specific molecular features or signals, future studies utilizing single-cell sequencing may provide novel insights of breast cancer TMEs.

In conclusion, high TMB alone does not strongly correlate with immune infiltrate or immune-related gene signatures in further unselected MBC. In our dataset, TMB-H predicted a more immune responsive microenvironment compared to TMB-L in HR+, HER2- tumors which could further be enhanced when selecting PD-L1+ tumors. This subset of patients would be relatively rare, though a small prospective trial assessing immunotherapy strategies in this population would be warranted. *B2M* mutation and *CD274* amplification may help predict benefit to ICI within TMB-H MBC. Co-occurring biomarkers within TMB-H breast cancer warrant further evaluation in larger cohorts for response or resistance to ICI to help develop composite predictive biomarkers in MBC.

## Data availability statement

The datasets presented in this article are not readily available because the raw data is protected proprietary information. Requests to access the datasets should be directed to aelliott@carisls.com of Caris Life Sciences.

## Ethics statement

Ethical review and approval was not required for the study on human participants in accordance with the local legislation and institutional requirements. Written informed consent for participation was not required for this study in accordance with the national legislation and the institutional requirements.

## Author contributions

SS and ET: Conception, design, data analysis, manuscript writing and editing. AE: Conception, design, data analysis, biostatistical analysis. RB-S and ST: data analysis, manuscript editing. SC, AT, and GS: data analysis, manuscript editing. All authors contributed to the article and approved the submitted version.
